# Healthcare workers’ willingness to respond following a disaster: a novel statistical approach toward data analysis

**DOI:** 10.1186/s12909-019-1561-7

**Published:** 2019-05-03

**Authors:** Stav Shapira, Michael Friger, Yaron Bar-Dayan, Limor Aharonson-Daniel

**Affiliations:** 1 PREPARED Center for Emergency Response Research, Ben-Gurion University of the Negev, P.O. Box 653, Beer-Sheva, Israel; 2 School of Public Health, Faculty of Health Sciences, Ben-Gurion University of the Negev, P.O. Box 653, Beer-Sheva, Israel; 3 Department of Public Health, Faculty of Health Sciences, Ben-Gurion University of the Negev, P.O. Box 653, Beer-Sheva, Israel

**Keywords:** Health personnel, Disaster planning, Statistical models, Earthquakes, Absenteeism

## Abstract

**Background:**

The willingness of healthcare workers (HCW) to respond is an important factor in the health system’s response capacity during emergencies. Although much research has been devoted to exploring this issue, the statistical methods employed have been predominantly traditional and have not enabled in-depth analysis focused on absenteeism-prone employees during emergencies. The present study employs an innovative statistical approach for modeling HCWs’ willingness to respond (WTR) following an earthquake.

**Methods:**

A validated questionnaire measuring knowledge, perceptions, and attitudes toward an earthquake scenario was distributed among Israeli HCWs in a hospital setting. Two regression models were employed for data analysis – a traditional linear model, and a quantile regression model that makes it possible to examine associations between explanatory variables across different levels of a dependent variable. A supplementary analysis was performed for selected variables using broken line spline regression.

**Results:**

Females under the age of forty, and nurses were the most absenteeism-prone sub-groups of employees (showed low WTR) in earthquake events. Professional commitment to care and perception of efficacy were the most powerful predictors associated with WTR across all quantiles. Both marital status (married) and concern for family wellbeing, designated as statistically significant in the linear model, were found to be statistically significant in only one of the WTR quantiles (the former in Q10 and the latter in Q50). Gender and number of children, which were not significantly associated with WTR in the linear model, were found to be statistically significant in the 25th quantile of WTR.

**Conclusions:**

This study contributes to both methodological and practical aspects. Quantile regression provides a more comprehensive view of associations between variables than is afforded by linear regression alone. Adopting an advanced statistical approach in WTR modeling can facilitate effective implementation of research findings in the field.

**Electronic supplementary material:**

The online version of this article (10.1186/s12909-019-1561-7) contains supplementary material, which is available to authorized users.

## Background

Earthquakes are liable to become mass casualty events [1, 2]. Healthcare workers (HCW) in both the pre-hospital and hospital settings are key players in the response to such events. They are required to provide lifesaving medical treatment to an influx of patients, possibly in the face of shortages in resources such as medical supplies and qualified manpower [3–6]. The State of Israel has contended with numerous mass casualty events caused by terrorism [7], but has little experience in dealing with natural disasters. Israel is located in a seismic risk area, and local experts warn that a powerful earthquake could well occur in the near future [8]. Enhancing the preparedness of its healthcare institutions and staff to deal with such a scenario is therefore seen as being of vital importance.

The clinical staff is directly responsible for fulfilling the core mission of the hospital – providing quality patient care – and is thus one of its key assets [9–11]. This is true for routine care and even more so during mass casualty events that call for patient care surge capacity [5, 12]. Absenteeism of essential HCWs during such events may result in delays in providing medical aid for casualties, increased morbidity, unnecessary complications, and possibly even loss of life [3, 13]. Much research has been devoted to exploring HCWs’ willingness to respond (WTR) during and following public health emergencies, and great efforts have been made to reduce absenteeism [14–16]. Studies from the field of human resource management suggest that developing individual and collective competencies among core employees and addressing their needs during emergencies can prevent absenteeism and enhance the organization’s resilience and capacity to contain such events [17, 18].

Three comprehensive literature reviews have sought to identify personal-, professional-, organizational-, and threat-related factors associated with WTR among HCWs following a public health disaster. One review focuses exclusively on pandemic influenza [19] while the other two relate to ‘disasters’ in general [20, 21]. Careful scrutiny of the results reveals conflicting findings regarding most factors. Only a few factors are in broad consensus, e.g. gender. Among the personal-related factors, the findings regarding gender are consistent and indicate that women tend to have a lower WTR than men [19, 20]. Concern for family, loved ones, pets, and personal obligations such as childcare and eldercare emerge as powerful barriers against WTR [19–21]. Among the professional-related factors, the greater part of the evidence indicates that clinicians have higher WTR than non-clinicians [19], but at the same time that nurses have low WTR compared with other clinicians [19]. Belief in duty to care, knowledge, perception of efficacy, and levels of training are described as facilitators of WTR [19–21]. Organizational-related factors are not discussed at length, and it seems that confidence in the employer’s ability to meet basic needs of employees (e.g. food, water, etc.) and other concerns is the primary factor in this category [19, 21]. The findings regarding threat-related factors demonstrate that WTR rates are lower for events that pose higher personal risk to employees, such as infectious diseases and other biological, chemical, and radiological hazards, as compared with natural disasters and technological or other man-made threats [19, 20]. This was also supported in a more recent study [22].

Although an extensive body of literature exists in this field, data analysis approaches have tended to be traditional, usually involving multivariate analysis based on linear or logistic regression. Consequently, the findings merely reflect general associations and cannot lead to identification of specific factors and characteristics associated with distinct levels of WTR – information that is essential for ameliorating preparedness. WTR is usually measured using an ordinal scale (i.e. Likert scale). The use of linear regression is considered less than optimal when analyzing ordinal data since the latter cannot be assumed to be normally distributed; nevertheless the use of this method is prevalent in WTR modeling [23] and exposes the findings to possible criticism [24]. Another common method is dichotomizing ordinal data in order to apply logistic regression analysis [25, 26], but this entrains loss of data, may lead to reduction in statistical power, and is generally not recommended [27]. A more flexible approach toward ordinal data analysis focused on the entire distribution of the dependent variable would provide far more useful and reliable information [28]. A possible candidate is quantile regression (QR) modeling. The use of QR was first introduced in economics [29], but it has since become popular in fields and disciplines such as medicine and health care [30, 31], and recently in ecological research and community resilience modeling [32]. QR enables division of the dependent variable into homogeneous segments (much like the structure of original data), and the relationship with independent variables is assessed separately for each of the segments (quantiles). Thus, no loss of data occurs, and a holistic view of the results is made possible. While QR is concerned with modeling the dependent variables, attention should also be paid to the modeling methods used for the independent variables. Effect size measures (e.g. regression coefficient and odds ratio) provide information regarding the direction and strength of association, but fail to identify the exact points across the distribution of continuous explanatory variables at which a statistically significant change occurs in the outcome variable. This is not an issue if the nature of the association is linear, but may constitute a limitation in more complex relationships [33]. Spline regression is a smoothly joined piecewise regression that addresses this issue by performing division of a continuous independent variable into linear segments connected to each other at their edges (called a knot). The model makes it possible to test whether a statistically significant change in the regression line slope occurs above and below a specific knot location. The properties of this regression fit complex distribution patterns, enabling examination of both shape and strength of association [28, 34].

Applying these analysis methods in WTR modeling can facilitate characterization of absenteeism-prone sub-groups – i.e. identification of personal characteristics related to HCWs with low levels of WTR – as well as in-depth examination of the relationships between professional and organizational factors across different levels of WTR.

The objectives of this study were to identify the personal and professional characteristics of HCWs with low levels of WTR following an earthquake by using a novel statistical approach and help develop strategies to ameliorate their willingness.

## Methods

### Survey instrument

A structured self-administered anonymous questionnaire was designed to assess HCW demographic characteristics, WTR, attitudes, perceptions, and knowledge level regarding an earthquake scenario (see Additional file 1). The questionnaire was a modified version of a validated tool used to measure preparedness components among HCWs in various emergency situations, and was adjusted to an earthquake scenario. Additional information describing the survey design and validation process is detailed in previous studies [23, 35, 36]. Information pertaining to demographics included gender, age, marital status, number of children residing at home, and professional role. Knowledge regarding different components of hospital response to an earthquake was measured through twelve multiple-choice questions, based on a national standardized operating procedure (SOP) formulated by the Israel Ministry of Health. To understand HCW perceptions, seven questions were asked concerning self- and organizational efficacy in an earthquake scenario (α = .87), using a 7-point Likert-type scale ranging from 1 (I strongly disagree) to 7 (I strongly agree). WTR following an earthquake was assessed using two items that dealt with personal WTR, as well as the perceived willingness of colleagues. Possible barriers (2 items related to concern for family wellbeing and damage to house due to the earthquake) and facilitators (2 items related to professional commitment to care for the injured or ill, and fear of losing place of employment) of WTR were also assessed. The items in this section were also rated on a 7-point Likert-type scale. The questionnaire was pre-tested and piloted with a convenience sample of 27 HCWs who were similar in their characteristics to the study population. Based on the participants’ recommendations, minor modifications were made in the final version.

### Study design and data collection

This cross-sectional survey study was conducted from November 2013 through June 2014 in all secondary and tertiary hospitals in Israel (*n* = 24). The study population included clinical health professionals (i.e. physicians, nurses and paramedical staff) employed in the five hospital departments likely to be the most involved in providing treatment to earthquake casualties: the emergency department; internal medicine division; surgical wards; orthopedics; and operating rooms.

Sample size was determined before study initiation. Based on previous findings indicating that levels of WTR to different emergency situations among HCWs ranged between 58 and 72% [26, 37, 38], a mean value of 65% participants reporting high levels of WTR (ranking between 5 and 7 on a Likert scale) was assumed for calculating the current sample size. A margin of error of 5% and a 99% confidence interval were taken in account, and accordingly a sample of 604 HCWs was sought. In expectation of a relatively low response rate to the surveys, especially among hospital personnel, it was decided to increase the sample size to 2400 HCWs (anticipating a response rate of 25%).

Distribution of the survey and allocation of participants were carried out in several steps. First, the research team met with hospital emergency managers in the Emergency Division of the Israel Ministry of Health. After the team’s objectives and study protocol had been presented, each hospital representative was given 100 questionnaires and asked to distribute twenty to the head nurse in each of the five departments mentioned. The head nurses also received a guideline sheet containing information about the study and detailed instructions regarding how and to whom to circulate the questionnaires (to six physicians, eight nurses, and six paramedical staff employed in their department; selection of specific participants in each category was according to the head nurse’s choice). Next, the completed questionnaires were collected by the head nurse within 2 weeks from distribution and returned to the hospital’s emergency manager. They were then sent back to the Ministry of Health and collected by the researchers. The importance of taking part in the survey was stressed at all levels by sending designated cover letters to hospital managers, heads of departments and head nurses and by including a personal appeal to the participants in the survey.

### Statistical analysis

Data analysis was performed in three main steps. In the first step, a univariate analysis with descriptive statistics was followed by a bivariate analysis conducted using Spearman’s correlation, chi-square test, Mann-Whitney U-test, Kruskal-Wallis one-way analysis of variance, and median test to estimate the associations between the dependent variable, namely WTR (defined as the mean score of the two items used to measure this element), and the other survey variables. In the second step. Two multivariate regression models (multivariate linear regression and multivariate quantile regression) were developed in order to identify associations between explanatory variables and WTR: gender, age, number of children residing at home, professional role, marital status, collected ‘knowledge’ score (number of correct answers), ‘efficacy’ measure, and four potential barriers and facilitators for WTR.

Linear regression modeling relies on finding a best-fitting regression line that minimizes the sum of squared errors of prediction. This simple and traditional model, which uses the mean values of data to calculate the regression line and is therefore sensitive to outliers, may not be an ideal method for capturing a central tendency of the data. Quantile regression, on the other hand, is a non-parametric method which consists of finding a regression line by minimizing the sum of the absolute residuals using the median rather than the mean value of the data across various quantiles of the dependent variable [39–41]. Thus, not only may quantile regression be expected to reflect the data in a more precise manner, it also illuminates associations between variables at different levels of the dependent variables and offers additional insights about the nature of these relationships [32].

Our analysis examined the regression coefficients across quantiles 5, 10, 25, 50 and 75 of the dependent variable (the 90th percentile was excluded due to paucity of observations).

In the third and final step, a supplementary analysis was performed to explore the variance of the WTR measure over the age distribution of participants using a broken line spline regression. To enhance the sensitivity of the results, the analysis was performed separately for females and males. Spline knots were fitted at ages 25, 30, 35, 40, 45, 50, 55, 60 and 64. The model was arrayed in a hierarchical design: the first block included the variables age and profession; the second block added the spline knots and employed a forward stepwise selection strategy (inclusion and exclusion criteria set at *p* > .05 and *p* < .10, respectively); and the last block added statistically significant explanatory variables identified in the previous multivariate analysis described above (professional commitment and efficacy). A *P* value of <.05 was considered statistically significant.

Data were analyzed using IBM Statistical Package for the Social Sciences (version 23.0; IBM corp, Armonk, NY) and STATA software (version 12.1; StataCorp, College Station, TX).

## Results

### Participants

Of the 2400 questionnaires distributed to HCWs in the hospitals, a total of 852 were completed and returned, yielding a response rate of 35% (response rates ranged from 11 to 93% across hospitals). The demographic and professional characteristics of the participants are detailed in Table 1. The majority of participants were nurses, female, and married (78% of all nurses were female). The mean age was 43, and most participants had 1–3 children residing with them at the time of the survey.

**Table 1:** Demographics, professional characteristics and knowledge regarding hospital response to an earthquake of participants (*n* = 852)^a^

	Demographics		N (Total) = 852	
			N (%)	
1	Gender	Female	532 (62)	
		Male	320 (38)	
2	Age (years): Mean (SD)		43.3 (11.2)	
3	Marital Status	Single	122 (14.5)	
		Married/Common law	640 (76.5)	
		Divorced/Widowed	72 (8.6)	
		Other	3 (0.4)	
4	Number of children residing in household (Under 17 years of age): Median (Interquartile Range)		2 (1–3)	
5	Professional role	Physician	220 (26)	
		Nurse	499 (58.5)	
		Paramedical staff	133 (15.5)	
Knowledge				
			Correct	Incorrect
			N (%)	N (%)
Safety measures to protect immobile patients			392 (54)	333 (46)
Actions to protect self			307 (39)	484 (61)
Actions to be taken immediately after an earthquake			497 (61)	320 (39)
Safety measures with damaged infrastructures			568 (71)	234 (29)
Who is authorized to evacuate a department?			65 (8)	761 (92)
Patient registration procedure			487 (60)	326 (40)
Recommended treatment protocol for crush syndrome			258 (32)	549 (68)
Appropriate action when casualty with minor injury presents to hospital			460 (57)	348 (43)
Appropriate action when casualty with major injury presents to hospital			603 (74)	210 (26)
Appropriate action when an anxiety-stricken patient presents to hospital			656 (80)	161 (20)
Command, control and communication in hospital after an earthquake			673 (83)	141 (17)
Communication with external institutions after an earthquake			203 (26)	574 (74)
Average knowledge score (number of correct answers)			6 ± 2.4 (MD = 6, 0–11)^b^	

^a^Without missing values; the rate of missing values ranged from 1 to 5% for the different variables

^b^Out of brackets – mean ± SD; In brackets – median, minimum-maximum

#### Univariate and bivariate analysis of study measures

Gender and profession were significantly associated with WTR. Females had significantly lower levels of WTR compared with male participants, as represented by median score (MD) and interquartile range (IQR): MD = 5 (IQR = 4–6) vs. MD = 5.5 (IQR = 5–6.5); *p* < .001, respectively. Nurses had significantly lower levels of WTR compared with physicians: MD = 5 (IQR = 4–6) vs. MD = 5.8 (IQR = 5–6.5); *p* > .001, respectively. Age was positively correlated with WTR (r_s_ = .14, p < .001). Other demographic characteristics did not reach statistical significance (*p* > .05).

Knowledge was measured through twelve multiple choice questions. An average knowledge score was calculated based on the participants’ number of correct answers. Participants’ responses are detailed in Table 1. The median knowledge score of participants was relatively low: MD = 6 (IQR = 5–8). When examined per profession, nurses had the highest knowledge score, namely MD = 7 (IQR = 6–8), versus MD = 6 (IQR = 4–8) for physicians and MD = 5 (IQR = 3–6.5) for paramedical staff (*p* < .001). Four knowledge questions had a correct response rate lower than 50%. These items related to: appropriate actions to protect oneself during an earthquake (39%); indicating the person authorized to evacuate a department following an earthquake (8%); familiarity with the treatment protocol of a common earthquake-related injury (32%); and communication procedures with external institutions or organizations (e.g. EMS services) following an earthquake (26%).

Efficacy in an earthquake scenario was measured using seven items related to self- and organizational efficacy. An efficacy measure was calculated based on the mean of ratings of all seven items. The median efficacy score was 4.8 (IQR = 4–5.5) (out of 7), similar to its sub-dimensions scores of self-efficacy: MD = 4.6 (IQR = 3.5–5.5) and organizational efficacy MD = 5.2 (IQR = 4–6). When examined per profession, nurses demonstrated a significantly higher rate of efficacy, namely MD = 5.1 (IQR = 4.3–5.7), as compared with MD = 4.5 (IQR = 3.4–5.5) for physicians and MD = 4.3 (IQR = 3.3–5.2) for paramedical staff (*p* < .001).

Willingness to respond to an earthquake scenario was measured through two items (personal WTR and perceived WTR of colleagues). Seventy six percent of participants reported they had “high WTR” (rating between 5 and 7 on a Likert scale). Only 72% of nurses reported high WTR, significantly lower compared with 82% among physicians and 80% among paramedical staff (*p* = .02).

‘Concern for family wellbeing’ and ‘Professional commitment to care for the injured or ill’ were reported by the largest number of participants as factors that could strongly affect the decision to report to work (91 and 92% of participants, respectively, rated these items between 5 and 7 on a Likert scale), followed by “Concern for house possibly damaged in the quake” (67%) and “Fear of losing place of employment” (34%). A significantly higher proportion of nurses (94%) noted “Concern for family wellbeing” as a possible barrier to WTR compared with physicians (84%) and paramedical staff (90%) (*p* = .001). Similar results were demonstrated for “Concern for house possibly damaged in the quake” – 70% of nurses indicated this as a possible barrier, compared with only 57% among physicians and 65% among paramedical staff (*p* = .01). No statistically significant differences were demonstrated for the remaining two items (*p* > .05).

#### Multivariate analysis

**Fig. 1:**
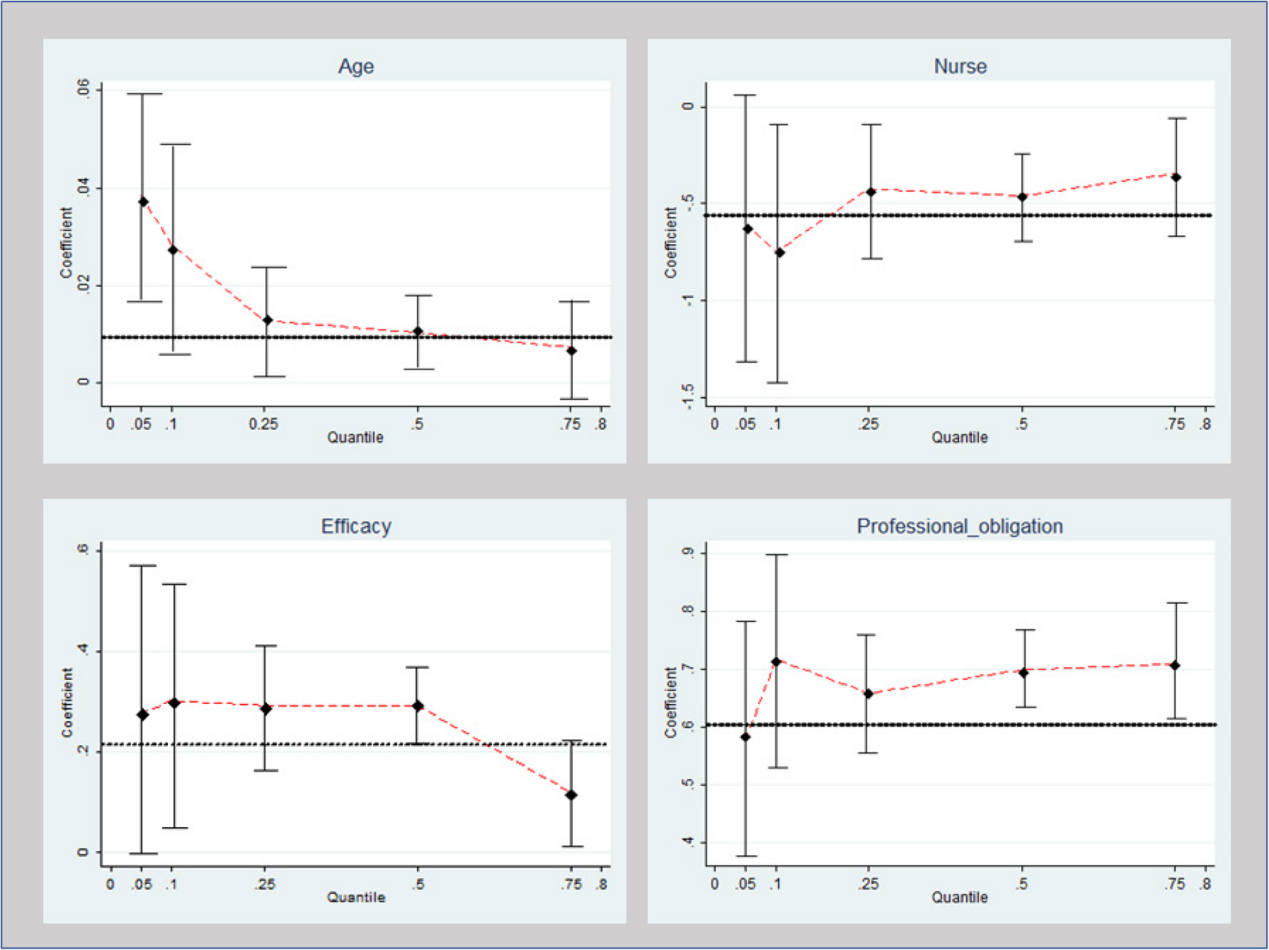
Quantile plots for ‘Willingness to respond’ displaying the statistically significant parameters in the multivariate regression analysis. The x-axis represents the location in the distribution (i.e. quantile) of the willingness measure; the y-axis represents the size of the unstandardized coefficient values at each point of the outcome distribution (controlling for all other variables). The black diamond-shaped markers represent the coefficient values across different quantiles; the red dashed line represents an estimation of coefficient values across a range of quantile distribution (Q5 to Q75); vertical lines (error bars) indicate 95% confidence interval; the horizontal black dashed line represents the unstandardized coefficient value in the linear regression (for each parameter)

Two multivariate regression models were employed to identify statistically significant associations between study variables and WTR. The linear regression results revealed six statistically significant predictors. Age (per year), beta (standardized regression coefficient) = .01, 95% CI: (.01, .02); efficacy score, beta = .24, 95% CI: (.16, .31); professional commitment, beta = .61, 95% CI: (.53, .68); being a nurse (compared with paramedical staff), beta = −.46, 95% CI: (−.70, −.22); being married (compared with being single), beta = −.25, 95% CI: (−.49, −.03); and ‘concern for family’s wellbeing’, beta = −.11, 95% CI: (−.18, −.03) had significant predictive value for WTR (see Table 2). The quantile regression results indicated statistically significant associations regarding eight predictors (see Table 2): gender, age, number of children, being a nurse, being married, efficacy score, concern for family’s wellbeing, and professional commitment. Gender was statistically significant only in Q25. Age was statistically significant across Q5 to Q50, the effect size being strongest in Q5 and decreasing gradually thereafter. Number of children was statistically significant only in Q25. Being a nurse was statistically significant in Q10 to Q75, the strongest effect size occurring in Q10. Being married was significant only in Q10. Concern for family’s wellbeing was statistically significant but only in Q50. Efficacy score and professional commitment were statistically significant across all quantiles of WTR. Figure 1 presents the statistically significant covariates (found to be statistically significant in more than one quantile) and the change in their effect size across various quantiles of WTR.

**Table 2:** Unstandardized coefficient estimates for linear regression analysis and quantile regression analysis (selected quantiles)

Variable		Linear Regression Coefficient (*p* value)	Quantile Regression – Coefficient (*p* value)				
			5th	10th	25th	50th	75th
Gender (female)		.15 (*p* = .12)	.20 (*p* = .55)	.42 (*p* = .06)	**.35 (*p*** **= .01)**	.07 (*p* = .43)	.13 (*p* = .28)
Age (per year)		**.01 (*p*** **< .001)**	**.04 (*p*** **= .01)**	**.03 (*p*** **= .01)**	**.01 (*p*** **= .02)**	**.01 (p = .01)**	.01 (*p* = .11)
No. of children		−.05 (*p* = .16)	−.01 (*p* = .85)	−.03 (*p* = .70)	**−.09 (*p*** **= .04)**	−.03 (*p* = .25)	−.05 (*p* = .16)
Profession (Paramedical staff)	Physician	−.06 (*p* = .66)	−.30 (*p* = .50)	−.23 (*p* = .46)	−.06 (*p* = .72)	.10 (*p* = .37)	−.09 (*p* = .57)
	Nurse	**−.46 (*p*** **< .001)**	−.66 (*p* = .12)	**−.71 (*p*** **= .02)**	**−.42 (*p*** **= .01)**	**−.45 (*p*** **< .001)**	**−.31 (*p*** **= .03)**
Marital status (Single)	Married	**−.25 (*p*** **= .04)**	−.58 (*p* = .15)	**−.55 (*p*** **= .04)**	−.21 (*p* = .21)	−.15 (*p* = .19)	−.01 (*p* = .98)
	Divorced	−.02 (*p* = .91)	−.43 (p = .50)	−.40 (*p* = .35)	.12 (*p* = .62)	.15 (p = .37)	.19 (*p* = .38)
	Other	−.18 (*p* = .78)	.20 (*p* = .73)	−.36 (p = .50)	−.83 (*p* = .08)	.34 (*p* = .51)	.28 (p = .50)
Knowledge score (% correct)		−.01 (*p* = .68)	−.03 (*p* = .96)	−.01 (*p* = .84)	−.02 (*p* = .40)	−.01 (*p* = .64)	.01 (*p* = .79)
Efficacy score (average)		**.24 (*p*** **< .001)**	**.35 (*p*** **= .02)**	**.30 (*p*** **= .01)**	**.30 (*p*** **< .001)**	**.28 (*p*** **< .001)**	**.13 (*p*** **= .01)**
Concern for family		**−.11 (*p*** **= .01)**	−.16 (*p* = .24)	−.14 (*p* = .16)	−.10 (*p* = .09)	**−.07 (*p*** **= .05)**	−.08 (*p* = .06)
Concern for house		.02 (*p* = .42)	−.02 (*p* = .82)	.01 (*p* = .97)	−.01 (*p* = .67)	−.03 (p = .28)	.04 (*p* = .25)
Professional commitment to care for injured or ill		**.61 (*p*** **< .001)**	**.59 (*p*** **< .001)**	**.69 (*p*** **< .001)**	**.65 (*p*** **< .001)**	**.71 (*p*** **< .001)**	**.71 (*p*** **< .001)**
Fear of losing place of employment		.02 (*p* = .40)	.04 (*p* = .61)	.06 (*p* = .25)	.06 (*p* = .09)	.01 (*p* = .46)	−.01 (*p* = .82)
Constant		.79 (*p* = .02)	−2.15 (*p* = .02)	−1.97 (*p* = .01)	−.40 (*p* = .37)	.06 (*p* = .83)	1.24 (*p* = .002)

Notes: Adjusted R^2^ for linear regression model = .35; adjusted R^2^ for quantile regression: 5th = .26; 10th = .27; 25th = .25; 50th = .24; 75th = .20

Reference groups for ‘gender’, ‘profession’, and ‘marital status’ are indicated in parentheses (female, paramedical staff, and single, respectively)

*p* values set in boldface indicate statistical significance (*p*<0.05)

#### Supplementary analysis – age and WTR (spline regression)

**Fig. 2:**
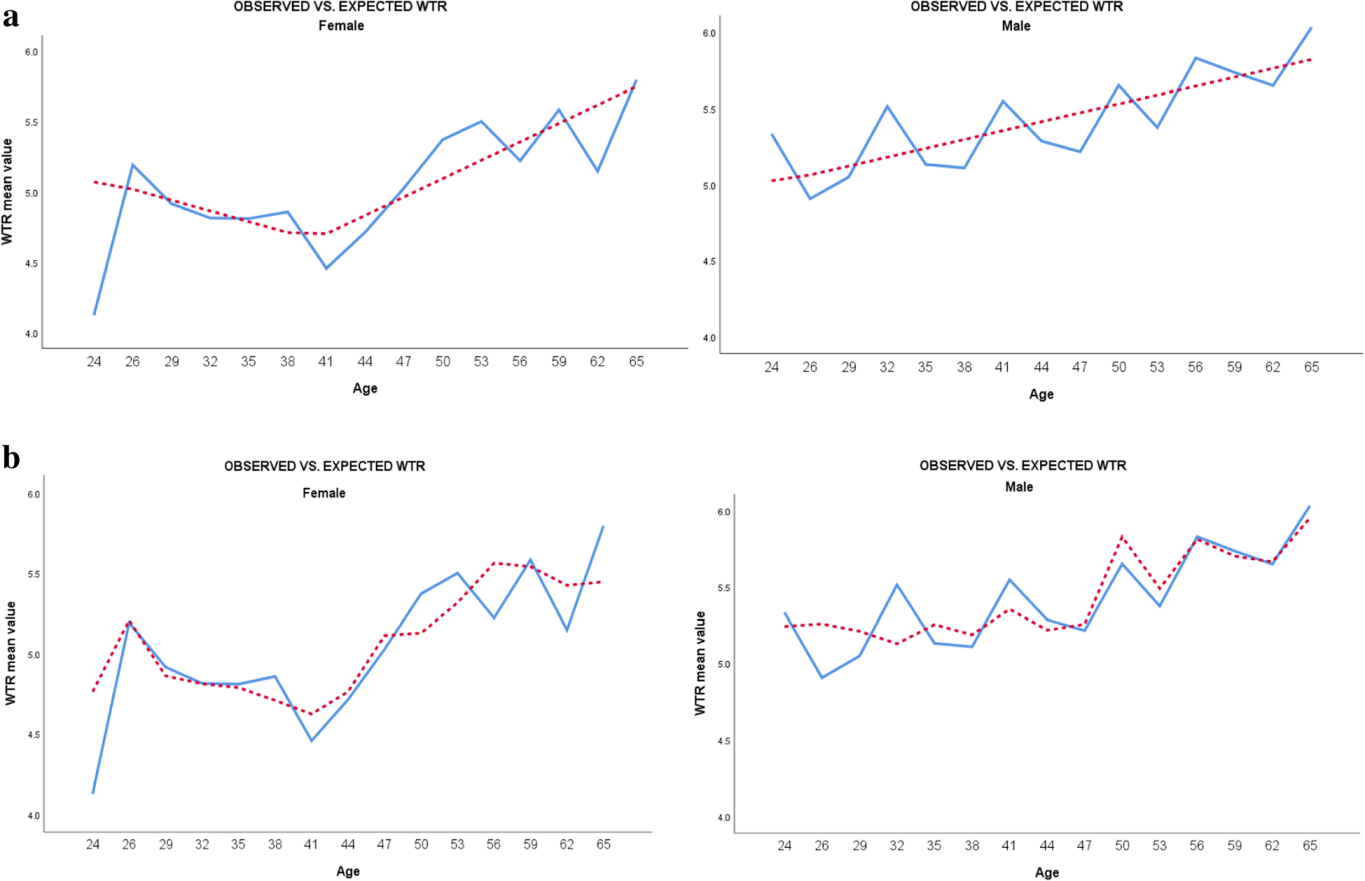
The relationship between participants’ age and mean value of willingness measure (WTR), according to gender (right column for males and left column for females); (a + b) describe the results of two multivariate spline regression models, the dependent variable being ‘willingness to respond’: (**a**) independent variables included in the model are age and spline age-based knots (X25, X30, X35, X40, X45, X50, X55, X60, X64); (**b**) independent variables included in the model are spline knots and age, profession, efficacy, and professional obligation. *The blue lines represent the observed values of WTR. Red dashed lines represent expected WTR.

To gain in-depth understanding of the relationship between the statistically significant demographic variable ‘age’ and WTR, an additional analysis was conducted using a linear regression model with spline. This analysis was conducted separately for females and for males. The results are described in Table 3 and Fig. 2.

**Table 3:** Results of multivariate linear spline regression (spline knots calculated for the variable ‘age’) per gender

Gender	Model			B (95% CI) Unstandardized coefficients	Beta Standardized coefficients	*p* value
Female (*n* = 529)	1	(Constant)		4.26 (3.70, 4.81)	–	**<0.001**
		Age		.020 (.01, .03)	.15	**<0.001**
		Profession (paramedical staff)	Physician	−.18 (−.67, .32)	−.03	.49
			Nurse	−.14 (−.47, .19)	−.04	.40
	2	(Constant)		5.73 (4.59, 6.88)	–	**<0.001**
		Age		−.02 (−.06, .01)	−.19	.14
		Age > 40		.07 (.02, .11)	.36	**.004**
		Profession (paramedical staff)	Physician	−.09 (−.59, .41)	−.02	.73
			Nurse	−.10 (−.43, .22)	−.03	.53
	3	(Constant)		1.34 (.21, 2.47)	–	**.02**
		Age		−.02 (−.04, .01)	−.13	.21
		Age > 40		.05 (.01, .09)	.25	**.01**
		Profession (paramedical staff)	Physician	.03 (−.39, .46)	.01	.87
			Nurse	−.34 (−.67, −.11)	−.12	**.007**
		Efficacy		.24 (.15, .34)	.20	**<0.001**
		Professional commitment		.54 (.45, .69)	.44	**<0.001**
Male (*n* = 318)	1	(Constant)		4.91 (4.19, 5.64)	–	**<0.001**
		Age		.02 (.01, .03)	.14	**.01**
		Profession (paramedical staff)	Physician	−.21 (−.70, .28)	−.07	.40
			Nurse	−.47 (−.98, .04)	−.16	.07
	2^a^	–		–		
	3	(Constant)		.58 (−.27, 1.43)	–	.18
		Age		.01 (−.01, .01	.04	.43
		Profession (paramedical staff)	Physician	−.21 (−.60, .19)	−.07	.30
			Nurse	−.75 (−1.16, −.34)	−.26	**<0.001**
		Efficacy		.20 (.08, .31)	.17	**.001**
		Professional commitment		.66 (.54, .78)	.52	**<0.001**

Note: the table describes the results of a hierarchical spline regression analysis (performed separately and identically for females and males). The first block included the variables age and profession; the second block added age-based spline knots. a stepwise selection was employed; the table includes only significant knots (if any); the last block added significant explanatory variables – professional commitment and efficacy

^a^The results for the males group did not reveal any statistically significant spline knots, thus, they were all excluded from the regression equation during the stepwise selection process

*p* values set in boldface indicate statistical significance (*p*<0.05)

The results of the analysis conducted for the female participants revealed that the fitted spline curve was statistically significant (*p* < .001). The slope took a significant turn at one knot: age > 40 (beta = .05, *p* = .01), meaning that WTR increases significantly among females older than forty. The results for the males group did not reveal any statistically significant spline knots, as they were all excluded from the regression equation during a stepwise selection process. This indicates a rather linear relationship between age and WTR among males.

## Discussion

Mitigating absenteeism is one of the most important challenges facing healthcare organizations in their struggle to improve response capabilities in the face of emergency and disaster situations. The present study sheds new light on the association of factors influencing WTR following an earthquake and discusses advanced statistical methods that might be applied for their identification and estimation.

The general high WTR rate (rating between 5 and 7 on a Likert scale) we found (76%) agrees with the rate reported in an earlier study, namely 78% WTR following an earthquake scenario; that study examined the response of hospital employees to various emergencies and disasters in the U.S. [42]. The WTR rate reported by nurses (70%) in the present study is higher than that cited in a previous Israeli study, in which only 57% of nurses indicated that they would report for duty following an earthquake [37]. This difference may be attributable to the inclusion of nursing students in the earlier study and its relatively small sample size. The WTR rate reported for nurses in the present study is also higher than the actual rate of Japanese nurses reporting for work following the Great East Japan earthquake in 2011 (48%) [15]; however, the radiological hazard posed by that event may have affected the nurses’ decision to report.

A comprehensive examination of our results reveals statistically significant differences between HCWs with respect to WTR and other elements of earthquake preparedness and response depending on profession. Nurses exhibited the highest levels of knowledge and efficacy, but also the lowest levels of WTR (compared with physicians). Being a nurse (in comparison with paramedical staff) was found to be a significant predictor of WTR, especially in the case of participants with lower levels of WTR. Knowledge was not a significant predictor of WTR. These are interesting findings that call into question theories that link knowledge and efficacy as determinants of behavioral change – represented in this study by participants’ WTR following an earthquake [43, 44]. To some degree our findings also stand in contradiction with several empirical studies that report a positive association between knowledge levels, self-efficacy, and participation in education and training programs (knowledge- and efficacy-enhancing activities), on one hand, and WTR following an earthquake [37] or other menacing scenarios [19, 20, 26], on the other. Our results agree in part with two studies conducted in the US and one in the UK, which found that physicians were more likely to be willing to report for duty during a catastrophic event than nurses [45–47]. However, only one of the latter three studies directly examined earthquakes, making it difficult to compare their results with our own. One possible explanation for this disparity might be the higher incidence of nurses pointing to family wellbeing and safety of the house as possible barriers to WTR (as compared with physicians and paramedical staff). Concern for family and loved ones emerges as one of the most powerful barriers to WTR reported in the literature. This is confirmed by two literature reviews that examined factors associated with healthcare personnel WTR following disaster situations [20] and during an influenza public health emergency [19]. (Concern for damage to the house was not generally discussed in these earlier studies, as that barrier is specific to earthquake scenarios.) The fact that nurses reported such barriers more frequently may be cultural; it may reflect the fact that nursing is a profession with female dominance and that women are still perceived (even by themselves) as the ones primarily responsible for dealing with household and family matters.

A strong positive association was found between the participants’ efficacy and professional commitment to care for the injured and ill and their WTR; the two variables were also identified as significant predictors of WTR in both regression models and across all WTR levels (quantiles). These factors seem to overshadow other potential barriers, such as concern for the safety of the home and fear of losing one’s place of employment, neither of which was found to be statistically significant in any of the models. While professional obligation is perceived almost as a moral imperative by HCWs and has been identified as a powerful factor influencing WTR even in scenarios that pose a personal risk for the worker (e.g. infectious diseases) [19, 20], it also lies at the heart of a complex conflict with personal and family obligations (e.g. child or elder care) [21]. This conflict is reflected in the present finding that ‘number of children’ and ‘concern for family wellbeing’ are statistically significant predictors of WTR (although the quantile model did not identify them as significant at the lowest levels of WTR). The supplementary analysis also revealed that young females (< 40 of age), and nurses (both females and males) were the most prone to absenteeism groups in regard to WTR following an earthquake. Here too tensions between professional and personal considerations could be at play, since in Israel’s family oriented society most women in this age group have childcare obligations. Indeed, as noted above, rates of concern for family and the home were high among this group.

The present findings should be taken into account when designing programs and activities aimed at improving staff preparedness for earthquake situations, both at the stage of pinpointing ‘at-risk’ groups among those employees whose participation is regarded as vital and in the selection of contents for seminars, drills, etc. For example, our results revealed that staff had low-to-medium levels of knowledge about their hospital’s standard operating procedure following an earthquake. Areas of acute shortage of knowledge were also identified; it is recommended that these areas be targeted in future activities. Special emphasis should be placed on enhancing the employees’ sense of efficacy and professional commitment in the context of duty to care following an earthquake, but at the same time they should be encouraged to voice their concerns regarding potential barriers and conflicts [23]. The healthcare system should do everything in its power to minimize these conflicts and strengthen the moral grounds for duty to care by providing employees with solutions during emergencies, such as childcare centers in the hospital compound and other family support measures [48, 49]. Planning for an appropriate workforce to address these issues should be carried out well in advance. This is also noted in the World Health Organization recommendations for developing a public health response to emergencies [50].

To the best of our knowledge, this study is the first to suggest the use of advanced regression models (quantile and spline regression) to analyze data related to knowledge, attitudes, and beliefs among healthcare personnel in the context of emergency and disaster situations. Employing regression equations that perform analysis of variables by dividing them into more homogeneous sections enables assessment of complex (nonlinear) relationships and can increase the sensitivity of the results. Such a capability can be particularly valuable for organizational heads (e.g. hospital directors) as it provides a prognostic tool enabling the identification of vulnerable sub-groups of a certain population and determining the factors that may influence these groups [32, 51–54]. This can help target preparedness efforts, and improve their efficiency.

In the present analysis, being a nurse (in comparison with paramedical staff) and age were found to be significantly associated across almost all levels (quantiles) of WTR, in contradistinction with other personal characteristics (e.g. gender and number of children), which were significantly associated only in the 25th quantile of WTR (a relatively low level of WTR) and did not even reach statistical significance in the traditional linear model. Being married (in comparison with being single) and concern for family wellbeing were singled out as statistically significant predictors in the linear model, but in the quantile model the two were significantly associated only at Q10 (low level of WTR) and Q50 (medium level of WTR), respectively. Efficacy and professional commitment to care were found to be statistically significant factors associated with all levels of WTR. An additional analysis using broken line spline regression was performed to determine the point across the participants’ age distribution at which a statistically significant change occurred in WTR levels (females under/above forty). The use of quantile and broken line spline regressions provides a more comprehensive view of the relationships between variables and is especially important when the source of interest lies at the ends of the distribution of the dependent variable (characterizing, for instance, HCWs with low WTR) [51, 52].

The present study demonstrates that the adoption of robust non-parametric regression analysis methods for exploring and characterizing a diverse population group such as healthcare employees provides insights that would not have been revealed had a traditional analysis been used. The implications of these findings go far beyond the present context of emergency and disaster preparedness. The use of a statistical approach that enables in-depth investigation of a particular variable of interest at all levels of the distribution makes it easier to translate research findings into practical and effective measures in the field. In this study, our ultimate purpose was to make vital information available to the heads of relevant authorities – hospital directors, department heads and emergency officers – in order to support decision and policy making aimed at improving preparedness and response capabilities in a future earthquake scenario. However, this approach can also be utilized to explore other issues related to organizational culture and the behavior of healthcare personnel, whether during routine operation or in emergencies.

The present study has certain limitations. One is that it relies on self-reported data regarding intention to respond following an earthquake. Another is that the analysis does not encompass administrative and ancillary staff, who also have an important role to play in the hospital’s response to earthquake events and who may present their own unique characteristics. The relatively low response rate in the current study may also pose a certain limitation, as it has implications for potential nonresponse bias, which may affect generalizability of study results. However, this was anticipated and taken under consideration while planning the data collection process and calculating sample size. Further research is needed along two fronts, first to explore and characterize the entire hospital workforce, and second to examine the proposed statistical approach in the context of other emergency and disaster scenarios, each posing its own distinct threats and risks to hospital personnel.

## Conclusions

The present study shows that the use of quantile regression and broken line spline regression analysis for modeling healthcare workers’ knowledge, attitudes, beliefs and intended behavior in the context of a future disaster provides a more comprehensive and sensitive view of the data than can be achieved by traditional regression analysis. These advanced analysis strategies can promote our understanding of the issues investigated and facilitate effective implementation of research findings in the field. We recommend adoption of the statistical approach presented in this study for analysis of issues related to the organizational behavior of healthcare personnel. In the context of an anticipated earthquake scenario, this can help healthcare institutions improve their preparedness efforts, ultimately enhancing response capabilities.

## Additional file

Additional file 1: An English version of the survey instrument (DOC 67 kb)

## Abbreviations

HCW: Health care workers
QR: Quantile regression
WTR: Willingness to respond

## References

[1] Blaikie P, Cannon T, Davis I, Wisner B. At risk: natural hazards, people’s vulnerability and disasters. Routledge; 2014.

[2] Shapira S, Aharonson-Daniel L, Shohet IM, Peek-Asa C, Bar-Dayan Y (2015). Integrating epidemiological and engineering approaches in the assessment of human casualties in earthquakes. Nat Hazards.

[3] Schultz CH, Koenig KL, Noji EK (1996). A medical disaster response to reduce immediate mortality after an earthquake. New Engl J Med.

[4] Kaji A, Koenig KL, Bey T (2006). Surge capacity for healthcare systems: a conceptual framework. Acad Emerg Med.

[5] Paturas J, Smith D, Smith S, Albanese J (2010). Collective response to public health emergencies and large-scale disasters: putting hospitals at the core of community resilience. J Bus Contin Emer Plan.

[6] Timbie JW, Ringel JS, Fox DS, Pillemer F, Waxman DA, Moore M (2013). Systematic review of strategies to manage and allocate scarce resources during mass casualty events. Ann Emerg Med.

[7] Adini B, Peleg K (2013). On constant alert: lessons to be learned from Israel’s emergency response to mass-casualty terrorism incidents. Health Aff.

[8] Levi T, Bausch D, Katz O, Rozelle J, Salamon A (2015). Insights from Hazus loss estimations in Israel for Dead Sea transform earthquakes. Nat Hazards.

[9] West Michael A., Borrill Carol, Dawson Jeremy, Scully Judy, Carter Matthew, Anelay Stephen, Patterson Malcolm, Waring Justin (2002). The link between the management of employees and patient mortality in acute hospitals. The International Journal of Human Resource Management.

[10] West Michael A., Guthrie James P., Dawson Jeremy F., Borrill Carol S., Carter Matthew (2006). Reducing patient mortality in hospitals: the role of human resource management. Journal of Organizational Behavior.

[11] Kabene SM, Orchard C, Howard JM, Soriano MA, Leduc R. (2006). The importance of human resources management in health care: a global context. Hum Resour Health 2006;4(1):20. 10.1186/1478-4491-4-20 PMC1552082 16872531

[12] Hick JL, Hanfling D, Burstein JL, DeAtley C, Barbisch D, Bogdan GM (2004). Health care facility and community strategies for patient care surge capacity. Ann Emerg Med.

[13] Bartels SA, VanRooyen MJ (2012). Medical complications associated with earthquakes. Lancet..

[14] Mitchell R, Ogunremi T, Astrakianakis G, Bryce E, Gervais R, Gravel D (2012). Impact of the 2009 influenza a (H1N1) pandemic on Canadian health care workers: a survey on vaccination, illness, absenteeism, and personal protective equipment. Am J Infect Control.

[15] Ochi S, Tsubokura M, Kato S, Iwamoto S, Ogata S, Morita T (2016). Hospital staff shortage after the 2011 triple disaster in Fukushima, Japan-an earthquake, tsunamis, and nuclear power plant accident: a case of the Soso District. PLoS One.

[16] Santos JR, Herrera LC, Yu KDS, Pagsuyoin SAT, Tan RR (2014). State of the art in risk analysis of workforce criticality influencing disaster preparedness for interdependent systems. Risk Anal.

[17] Lengnick-Hall Cynthia A., Beck Tammy E., Lengnick-Hall Mark L. (2011). Developing a capacity for organizational resilience through strategic human resource management. Human Resource Management Review.

[18] Lesperance A, Miller J. Preventing absenteeism and promoting resilience among health care workers in biological emergencies. U.S. Department of Energy. 2009. http://www.pnl.gov/main/publications/external/technical_reports/PNNL-18405.pdf. Accessed 15 Jan 2016.

[19] Devnani M (2012). Factors associated with the willingness of health care personnel to work during an influenza public health emergency: an integrative review. Prehosp Disaster Med.

[20] Chaffee M (2009). Willingness of health care personnel to work in a disaster: an integrative review of the literature. Disaster Med Public Health Prep.

[21] Connor SB (2014). When and why health care personnel respond to a disaster: the state of the science. Prehosp Disaster Med.

[22] Charney RL, Rebmann T, Flood RG (2015). Hospital employee willingness to work during earthquakes versus pandemics. J Emerg Med.

[23] Shapira S, Aharonson-Daniel L, Bar-Dayan Y, Sykes D, Adini B (2016). Knowledge, perception, attitudes, and willingness to report to work in an earthquake: a pilot study Canadian vs. Israeli hospital nursing staff. Int Emerg Nurs.

[24] Allen IE, Seaman CA (2007). Likert scales and data analyses. Qual Prog.

[25] Balicer RD, Omer SB, Barnett DJ, Everly GS (2006). Local public health workers' perceptions toward responding to an influenza pandemic. BMC Public Health.

[26] Balicer RD, Barnett DJ, Thompson CB, Hsu EB, Catlett CL, Watson CM (2010). Characterizing hospital workers' willingness to report to duty in an influenza pandemic through threat-and efficacy-based assessment. BMC Public Health.

[27] Norman G (2010). Likert scales, levels of measurement and the “laws” of statistics. Adv Health Sci Educ.

[28] Harrell F. Regression modeling strategies: with applications to linear models, logistic and ordinal regression, and survival analysis. Springer; 2015.

[29] Koenker Roger, Bassett Gilbert (1978). Regression Quantiles. Econometrica.

[30] Yu K, Lu Z, Stander J (2003). Quantile regression: applications and current research areas. J R Stat Soc: Series D (The Statistician).

[31] Austin PC, Tu JV, Daly PA, Alter DA (2005). The use of quantile regression in health care research: a case study examining gender differences in the timeliness of thrombolytic therapy. Stat Med.

[32] Cohen O, Bolotin A, Lahad M, Goldberg A, Aharonson-Daniel L (2016). Increasing sensitivity of results by using quantile regression analysis for exploring community resilience. Ecol Indic.

[33] Marrie RA, Dawson NV, Garland A (2009). Quantile regression and restricted cubic splines are useful for exploring relationships between continuous variables. J Clin Epidemiol.

[34] Marsh LC, Cormier DR. Spline regression models (Vol. 137). Sage; 2001.

[35] Rokach A, Cohen R, Shapira N, Einav S, Mandibura A, Bar-Dayan Y (2010). Preparedness for anthrax attack: the effect of knowledge on the willingness to treat patients. Disasters..

[36] Schwartz D, Shapira S, Bar-Dayan Y (2014). Health care workers’ knowledge and confidence in personal protective equipment during the H1N1 pandemic in Israel. Disaster Med Public.

[37] Ben Natan M, Nigel S, Yevdayev I, Qadan M, Dudkiewicz M (2014). Nurse willingness to report for work in the event of an earthquake in Israel. J Nurs Manag.

[38] Balicer RD, Catlett CL, Barnett DJ, Thompson CB, Hsu EB, Morton MJ (2011). Characterizing hospital workers' willingness to respond to a radiological event. PLoS One.

[39] Gould W. Quantile regression with bootstrapped standard errors. Stata Tech Bull. 1993;2(9).

[40] Hao L, Naiman DQ. Quantile regression (No. 149). Sage; 2007.

[41] Koenker R. Quantile regression (No. 38): Cambridge university press; 2005.

[42] Cone DC, Cummings BA (2005). Hospital disaster staffing: if you call, will they come?. Am J Disaster Med.

[43] Bandura A (1997). Self efficacy: the exercise of control.

[44] Michie S, Johnston M, Francis J, Hardeman W, Eccles M (2008). From theory to intervention: mapping theoretically derived behavioural determinants to behaviour change techniques. Appl Psychol.

[45] Qureshi K, Gershon RR, Sherman MF, Straub T, Gebbie E, McCollum M (2005). Health care workers’ ability and willingness to report to duty during catastrophic disasters. J Urban Health.

[46] Irvin CB, Cindrich L, Patterson W, Southall A (2008). Survey of hospital healthcare personnel response during a potential avian influenza pandemic: will they come to work?. Prehosp Disaster Med.

[47] Damery S, Wilson S, Draper H, Gratus C, Greenfield S, Ives J (2009). Will the NHS continue to function in an influenza pandemic? A survey of healthcare workers in the West midlands, UK. BMC Public Health.

[48] Israel State Comptroller’s Report. The deployment of the hospitalization system for times of emergency and its functioning during the war. Jerusalem, Israel. 2007. http://www.mevaker.gov.il/he/Reports/Report_334/af06b204-d7ac-4cd2-ab50-b99d02a204eb/part07.pdf [Hebrew]. Accessed 8 Aug 2016.

[49] Iserson KV, Heine CE, Larkin GL, Moskop JC, Baruch J, Aswegan AL (2008). Fight or flight: the ethics of emergency physician disaster response. Ann Emerg Med.

[50] World Health Organization (2007). Ethical considerations in developing a public health response to pandemic influenza.

[51] Royston P, Ambler G, Sauerbrei W (1999). The use of fractional polynomials to model continuous risk variables in epidemiology. Int J Epidemiol.

[52] Gebregziabher M, Lynch CP, Mueller M, Gilbert GE, Echols C, Zhao Y (2011). Using quantile regression to investigate racial disparities in medication non-adherence. BMC Med Res Methodol.

[53] Fitzenberger B, Wilke RA. Quantile regression methods. Emerging Trends in the Social and Behavioral Sciences: An Interdisciplinary, Searchable, and Linkable Resource 2015;1–18.

[54] Bonanno GA, Romero SA, Klein SI (2015). The temporal elements of psychological resilience: an integrative framework for the study of individuals, families, and communities. Psychol Inq.

