# Supplementary material for: Healthcare workers’ willingness to respond following a disaster: a novel statistical approach toward data analysis
Source: BMC Med Educ. 2019 May 3;19:130. doi: 10.1186/s12909-019-1561-7 (PMC6499969; doi:10.1186/s12909-019-1561-7)
Supplement: Supplementary file 1 — An English version of the survey instrument (DOC 67 kb) [file 12909_2019_1561_MOESM1_ESM.doc]

**Appendix A**: An English version of the survey instrument

The following anonymous questionnaire aims to evaluate knowledge, perceptions and willingness to report to work among hospital personnel following an earthquake. If you choose to participate, do not write your name on the questionnaire.

The data gathered will be available only to the main investigators, and will be used solely for research purposes and improvement of emergency preparedness.

You are free to refuse to participate in this research project or to withdraw your consent and discontinue participation in the project at any time without penalty or loss of benefits to which you are otherwise entitled. Your participation will not affect your relationship with the hospital.

Your participation in this study is voluntary. ***Returning this survey implies your consent to participate in this research***

We sincerely thank you for your response and cooperation.

If you have any questions or concerns about completing the questionnaire or about being in this study, you may contact me at [stavshap@bgu.ac.il](mailto:stavshap@bgu.ac.il)

**Part A – Personal information**

1. **Gender**: 1) Male 2) Female

2. **Year of birth**: |___|___|_9_|_1_|

3. **Family Status**: 1- Single 2- Married 3- Divorced/Widowed 4- Other

4. **Number of children residing with you**: |___|___|

5. **Your main profession (occupation):**

A.□ Physician B.□ Nurse C. □ Paramedical occupation

6. **The main department in which you are employed**? (please circle)

1. Emergency Department
2. Surgical Ward
3. Internal Ward
4. Orthopedics
5. Operating Rooms

**Part B – Knowledge and perceptions**

**10. Perceptions of your role following an** earthquake:

|  |  | strongly agree | agree | tend to agree | I am impartial | tend to disagree | disagree | strongly disagree |
| --- | --- | --- | --- | --- | --- | --- | --- | --- |
| A. | **My** **role is vital** to my organization’s effective management of an earthquake |  |  |  |  |  |  |  |
| B. | **The hospital is prepared** to provide effective response to an earthquake |  |  |  |  |  |  |  |
| C. | I am **familiar with my role** in the hospital’s operation following an earthquake |  |  |  |  |  |  |  |

**11. Perceptions of knowledge and competency concerning an earthquake scenario:**

|  |  | strongly agree | agree | tend to agree | I am impartial | tend to disagree | disagree | strongly disagree |
| --- | --- | --- | --- | --- | --- | --- | --- | --- |
| A. | I **have sufficient knowledge** concerning treatment of earthquake victims |  |  |  |  |  |  |  |
| B. | I am familiar with the **hospital's standard operating procedure** for earthquakes |  |  |  |  |  |  |  |
| C. | I **feel safe to stay at the hospital** if an earthquake occurs |  |  |  |  |  |  |  |
| D. | I **feel that I am competent** as a caregiver to manage an earthquake |  |  |  |  |  |  |  |

12. Aligned with the Ministry of Health’s doctrine, what are the **protection measures** that should be provided for **immobile patients** during an earthquake?

A. Evacuate the patient with his bed to an external site outside the department

B. Evacuate the patient with his bed to the departmental protected area

C. There is no way to protect immobile patients

D. Protect the patients in their beds by placing a pillow over their heads

E. I don’t know

13. Aligned with the Ministry of Health’s doctrine, what are the **personal protective actions** the staff must implement during an earthquake?

A. Exit the department into the stairway, sit down, and protect the head

B. Take cover underneath a table/furniture or sit close to an internal wall, and protect the head

C. Depart externally, outside of the hospital's structure

D. Lie down on the ground and protect the head

E. Dependent on the floor you are present in during the earthquake

F. I don’t know

14. According to the standard operating procedure, what are the **immediate actions** to be implemented **immediately following the earthquake**?

A. Immediate evacuation of all patients from the department

B. Identification of hospital's departments that were damaged and provision of assistance as needed

C. Scout the area to locate casualties and damage; evacuate patients needing medical attention or resuscitation to the Emergency Department

D. Concentrate staff and patients in the department and wait for instructions from the
hospital management

E. I don’t know

15. In case of potential **damage to gas pipes and/or electricity supply infrastructure**, what security measures should be implemented?

A. An immediate evacuation of the department

B. The electricity, water, and gas supplies should be disconnected immediately

C. A substitute electricity source should be applied (a generator)

D. No action should be taken since the earthquake has passed

E. I don’t know

16. Who is **authorized** to issue an **evacuation** of a department/unit?

A. The hospital's management solely

B. The hospital's management or, in case there is no possibility to contact them – the most senior member of the department

C. The most senior member of the department

D. The head of the department

E. I don’t know

17. What **medical registration procedures** apply regarding admittance of patients to the hospital following an earthquake?

A. The routine medical registration will be continued

B. Manual registration will be applied and a list of casualties will be transferred to the operation center and the information center

C. A small card will be utilized for every patient stating basic details and diagnosis

D. In an emergency situation there is no time for an organized medical registration.

E. I don’t know

18. What is the recommended **treatment protocol** for a casualty suffering from **a crush syndrome** upon arrival at the hospital?

A. Amputation of the wounded limb

B. Fasciotomy and extensive debridement of the necrotic muscle

C. Aggressive treatment with fluids and diuretics to prevent systemic complications

D. Immediate hemodialysis

E. I don’t know

19. What is the appropriate action when a **lightly injured** casualty (for example: with limb wounds) presents to the hospital?

A. The patient should be directed to a designated site deployed outside the hospital
 area

B. The patient should enter the hospital area and provided with immediate treatment

C. The patient should be evacuated to a distant hospital for treatment

D. The patient should be directed to a designated site to be treated by a social worker /
 psychologist

E. I don’t know

20. What is the appropriate action when a **severely injured** person (for example, suffering from crush syndrome or needing amputation) presents to the hospital?

A. The patient should be directed to one of the designated sites deployed outside
 the hospital area

B. The patient should enter the hospital area and provided with immediate treatment

C. The patient should be evacuated to a distant hospital for treatment

D. The patient should be directed to a designated site to be treated by a social worker /
 psychologist

E. I don’t know

21. What is the appropriate action when an **anxiety-stricken** patient presents to the hospital?

A. The patient should be directed to one of the designated sites deployed outside
 the hospital area

B. The patient should enter the hospital area and provided with immediate treatment

C. The patient should be evacuated to a distant hospital for treatment

D. The patient should be directed to a designated site to be treated by a social worker /
 psychologist

E. I don’t know

22. Following an earthquake, how will the **control** **and communication** inside the hospital will be conducted?

A. No organized report mechanism can be implemented during an emergency

B. There is need to report solely to the director of the emergency department

C. An emergency operation center will be created by the management; reports to the center will be transferred through the routine communication systems (if operable) or through messengers

D. An emergency operation center will be created by the management and turned to only
 when needed

E. I don’t know

23. Immediately following an earthquake, collapse of **communication** mechanisms may occur between the hospital and external institutions. **Who should be reported to** during this time?

A. The regional EMS (emergency medical services) center via ambulance drivers and liaison officers

B. There is a need to wait for renewal of communication channels, and then a report
 should be submitted to the Ministry of Health

C. Media reporters (television, radio)

D. The Israeli police via local/field police teams

E. I don’t know

28. **Willingness to report to work following an earthquake:**

|  |  | Yes, without a doubt | Yes, almost positive | probably | I can’t decide | The chances are low | The chances are very low | No, I don’t believe they will show up |
| --- | --- | --- | --- | --- | --- | --- | --- | --- |
| A. | **Will you report to work** immediately after an earthquake? |  |  |  |  |  |  |  |
| B. | In your opinion, **will your colleagues report to work** immediately after an earthquake? |  |  |  |  |  |  |  |

29. **To what extent, in your opinion, will the following factors influence your decision to report to work following an earthquake?**

|  |  | To a very large extent | To a large extent | To a medium extent | To an undefinable extent | To a small extent | To a very small extent | Not at all |
| --- | --- | --- | --- | --- | --- | --- | --- | --- |
| A. | Concern for **my family’s wellbeing** |  |  |  |  |  |  |  |
| B. | Concern that **my house** will be damaged in the earthquake |  |  |  |  |  |  |  |
| C. | **Professional commitment** to care for the injured or ill |  |  |  |  |  |  |  |
| D. | Fear of **losing my place of employment** due to my absence |  |  |  |  |  |  |  |

**We thank you sincerely for your time and attention and your contribution for this important study.**
